# Deep Learning-Based DNA Methylation Detection in Cervical Cancer Using the One-Hot Character Representation Technique

**DOI:** 10.3390/diagnostics15172263

**Published:** 2025-09-07

**Authors:** Apoorva, Vikas Handa, Shalini Batra, Vinay Arora

**Affiliations:** 1 Department of Biotechnology, Thapar Institute of Engineering & Technology, Patiala 147004, India; vikas.handa@thapar.edu; 2 Department of Computer Science & Engineering, Thapar Institute of Engineering & Technology, Patiala 147004, India; sbatra@thapar.edu (S.B.); vinay.arora@thapar.edu (V.A.)

**Keywords:** DNA methylation, character representation technique, dimer encoding, deep learning, artificial intelligence, cervical cancer, machine learning, promoter methylation

## Abstract

**Background**: Cervical cancer is among the most prevalent malignancies in women worldwide, and early detection of epigenetic alterations such as Deoxyribose Nucleic Acid (DNA) methylation is of utmost significance for improving clinical results. This study introduces a novel deep learning-based framework for predicting DNA methylation in cervical cancer, utilizing a UNet architecture integrated with an innovative one-hot character encoding technique. **Methods**: Two encoding strategies, monomer and dimer, were systematically evaluated for their ability to capture discriminative features from DNA sequences. Experiments were conducted on Cytosine–Guanine (CG) sites using varying sequence window sizes of 100 bp, 200 bp, and 300 bp, and sample sizes of 5000, 10,000, and 20,000. Model validation was performed on promoter regions of five cervical cancer-associated genes: *miR-100*, *miR-138*, *miR-484*, *hTERT*, and *ERVH48-1*. **Results**: The dimer encoding strategy, combined with a 300-base pair window and 5000 CG sites, emerged as the optimal configuration. The proposed framework demonstrated better predictive performance, with an accuracy of 91.60%, sensitivity of 96.71%, specificity of 87.32%, and an Area Under the Receiver Operating Characteristic (AUROC) score of 96.53, significantly outperforming benchmark deep learning models, including Convolutional Neural Networks and MobileNet. Validation on promoter regions further confirmed the robustness of the model, as it accurately identified 86.27% of methylated CG sites and maintained a strong AUROC of 83.99, demonstrating its precision–recall balance and practical relevance during validation in promoter-region genes. **Conclusions**: These findings establish the potential of the proposed UNet-based approach as a reliable and scalable tool for early detection of epigenetic modifications. Thus, the work contributes significantly to improving biomarker discovery and diagnostics in cervical cancer research.

## 1. Introduction

**Figure 1:**
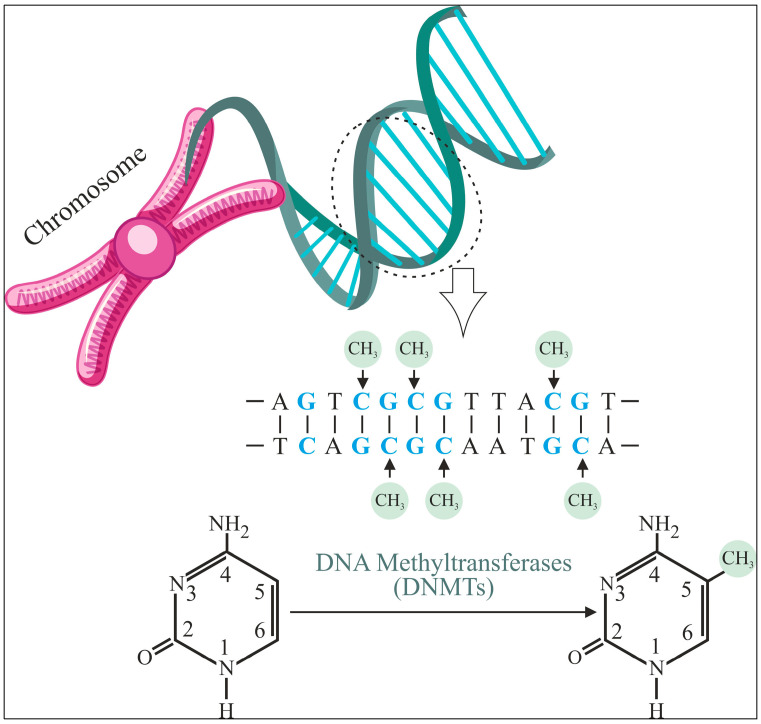
Schematic representation of the DNA methylation process.

Deoxyribonucleic Acid (DNA) methylation is a biochemical process that regulates many genes. It is a key epigenetic mark implicated in cell fate regulator silencing during normal growth and disease development. DNA methylation plays a crucial role in controlling X-chromosome inactivation, genomic imprinting, and gene expression in animals. As shown in Figure 1, this mechanism involves the addition of a methyl group at the fifth carbon of a Cytosine within Cytosine–Phospho–Guanine (CpG) dinucleotides, catalyzed by DNA Methyltransferases (DNMTs). DNA methylation patterns are intricately linked to cellular memory, lineage determination, and chromatin structure regulation [1]. Aberrations in these patterns, including promoter hypermethylation and global hypomethylation, have been associated with numerous diseases, including cancer, where these modifications play a pivotal role in tumorigenesis and genomic instability [2,3].

The potential of DNA methylation signatures as biomarkers for cancer diagnosis and prognosis has been highlighted in various recent research studies. However, despite substantial advancements in genome-wide methylation profiling enabled by Next-Generation Sequencing (NGS) technologies and publicly accessible datasets like ENCODE, challenges still persist in fully understanding the dynamics of DNA methylation, particularly in complex diseases such as cervical cancer [4,5]. The fourth most frequent malignancy in women worldwide is cervical cancer. It often arises due to consistent infection with high-risk Human Papillomavirus (HPV) strains [6]. These infections can induce DNA methylation alterations, contributing to malignant transformation in cervical epithelial cells [7].

Although Artificial Intelligence (AI) has improved epigenetic marker prediction in cancer cases, yet challenges still exist in discovering novel features for robust models. The current study presents a framework using statistical and deep learning methods to identify key methylation sites and refine existing biomarkers for cervical cancer prognosis. The approach has enhanced predictive accuracy, interpretability, and clinical relevance, supporting early detection and personalized treatment.

**Figure 2:**
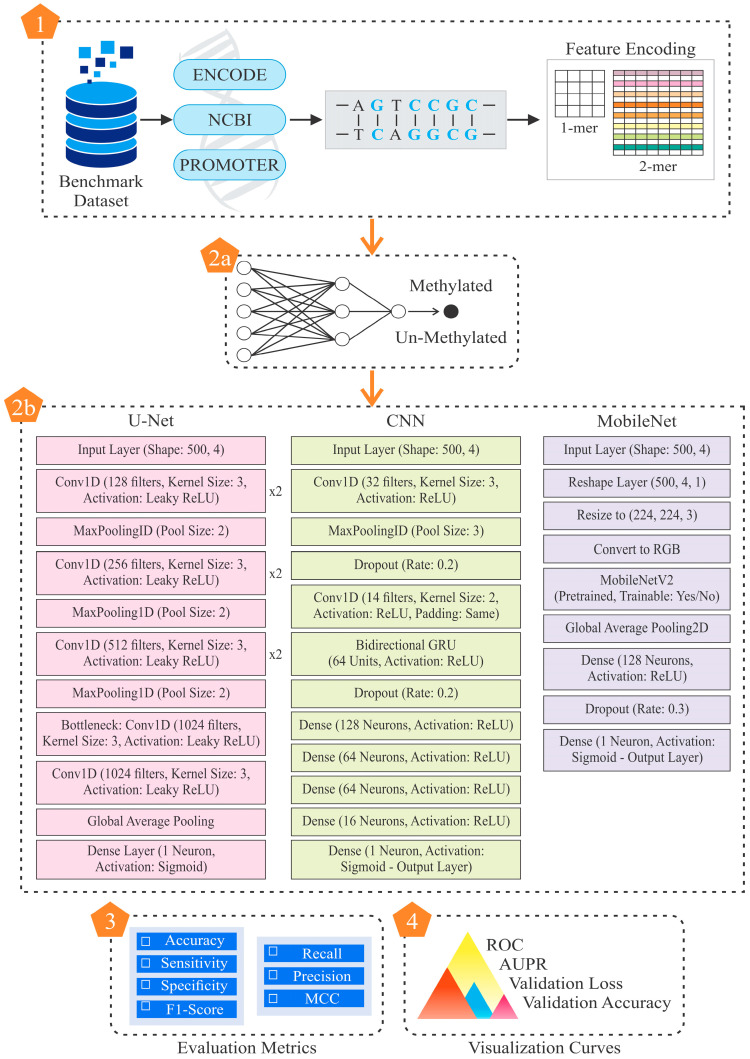
Description of the algorithm for detecting DNA methylation.

The existing methods are often standalone models that operate in a single domain, whereas hybrid systems operate in many domains and are capable of performing the classification process more efficiently. However, they are not commonly used in research on cervical cancer diagnosis. Thus, further research on DL-based computational diagnostic systems is required, since parameters related to CG methylation need to be quantitatively reliable in a number of sequential patterns. The proposed study highlights the importance of feature extraction, which may lead to understanding cervical cancer’s association with the underlying DNA pattern. The previous models, although quite useful, have shown opportunities for additional optimization regarding sensitivity, specificity, and overall accuracy. Thus, the current work is a modest attempt to improve upon the limitations of existing models. Figure 2 describes the comprehensive workflow of this study.

### Novel Contribution of the Study

This study introduces several methodological advancements and scientific insights that extend beyond existing approaches in DNA methylation prediction for cervical cancer. It presents a hybrid pre-processing and modeling framework for DNA methylation prediction in cervical cancer that is methodologically innovative and biologically meaningful. The model effectively balances local motif detection with broader contextual learning by employing an adaptive windowing strategy (100 bp, 200 bp, and 300 bp). To ensure robustness, scalability assessment was conducted across varying sample sizes (5000, 10,000, and 20,000 CpG sites), offering practical guidance for datasets with limited availability, such as rare disease cohorts. A key novel approach lies in the dual encoding scheme, which strategically integrates monomer and dimer representations to capture both first-order nucleotide information and higher-order dependencies, enhancing the discriminative capacity of features. Furthermore, empirical benchmarking highlights the comparative efficacy of encoding strategies, while gene-level validation on promoter regions of five cervical cancer–associated genes (*miR-100*, *miR-138*, *miR-484*, *hTERT*, and *ERVH48-1*) links computational predictions with clinically interpretable biomarkers. Collectively, these contributions advance epigenomic modeling by improving feature representation, contextual learning, and translational relevance.

## 2. Materials and Methods

### 2.1. Benchmark Dataset

Two publicly available databases, i.e., ENCODE and the National Center for Biotechnology Information (NCBI), were used for the purpose of this study to ensure the reliability and comprehensiveness of the research dataset [4,8]. High-throughput DNA methylation data from bisulfite-sequenced cervical cancer cell lines (HeLa) were acquired from the ENCODE database. The dataset included chromosomal locations and methylation percentages for 51,181 CpG sites derived from experimental results. A subset of 5000 CpG sites was selected from the dataset for computational efficiency and representative analysis. Due to the similarity in DNA methylation percentage patterns between this subset and the whole dataset, this subset was selected to substantially reduce the complexity of the training time. The detailed specifications and other information related to the ENCODE database are outlined as follows:

It has been accessible since 16 January 2011 and is identified by Lab ID: SL712 with subID-1152. This collection is catalogued under the GEO accession number GSM683828 and the UCSC accession number wgEncodeEH001363. These unique identifiers facilitate both the traceability and accessibility of the dataset, ensuring its utility for academic research and future reference. To complement the dataset mentioned above, DNA methylation data from normal human cells were sourced from the GenBank accession number CM000666.1 (GRCh37.p13/hg19) of the NCBI database. The integration of data from these two platforms enabled the construction of a curated dataset for this study, ensuring balanced representation and biological relevance.

HeLa, an immortalized cell line widely used in cancer research, provides comprehensive DNA methylation data across all 24 human chromosomes. To enhance the generalizability of the algorithm, chromosome 4 was selected for analysis due to its length being of medium size among all chromosomes, thereby avoiding bias from extremes in chromosome size. Each CpG site was analyzed using a sliding window approach with varying window sizes to maximize predictive performance. The methylation values of each CG site were categorized into two classes: methylated and unmethylated. The classification criterion, Beta (β), was used to define these categories. In this study, CG sites with methylation percentages of 50% or higher were classified as methylated, while those below this threshold were considered unmethylated. This cut-off value was selected due to its suitability for prediction, as supported by findings from previous studies [9,10,11,12,13,14]. A window size of 300 bp was determined to provide the highest accuracy in the proposed approach. Corresponding genomic sequences for the selected CpG sites, aligned with the chosen window size, were extracted from the aforementioned human genome assembly available through the NCBI database. This curated dataset served as the foundation for downstream feature extraction and model training, enabling robust analysis of methylation patterns.

### 2.2. Feature Encoding Methods

Two types of character encoding, i.e., monomer and dimer encoding, were applied for feature extraction [15]. One technique for feature engineering and data preparation that can convert discrete categorical variables into sparse binary codes is monomer encoding. The character encoding principle uses a variable with *n* categories to create a vector of length n, in which all the elements are zero and only one is 1. The position of one indicates the category of the variable [16]. Character encoding maps the four DNA bases ‘*A*’, ‘*G*’, ‘*C*’, and ‘*T*’ as shown below:

(1) $$\text{‘}A\text{’}\;:\left[1,\;0,\;0,\;0\right],\;\text{‘}C\text{’}\;:\left[0,\;1,\;0,\;0\right],\;\text{‘}G\text{’}\;:\left[0,\;0,\;1,\;0\right],\;\text{‘}T\text{’}\;:\left[0,\;0,\;0,\;1\right]$$

**Figure 3:**
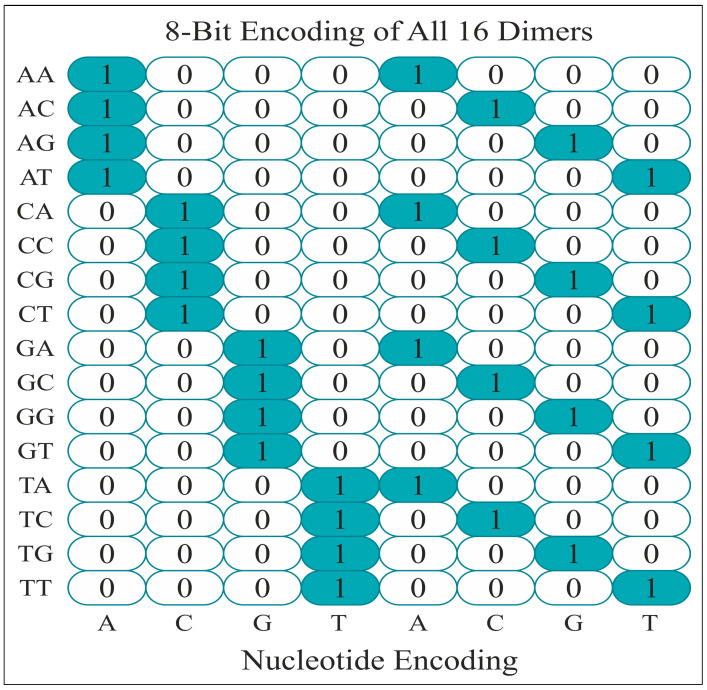
Feature encoding for 16 pairs of dinucleotides.

Similarly, the principle mentioned above was utilized to encode these DNA bases as dinucleotides, which gave 16 indices for 16 combinations of dinucleotides. A DNA sequence of 300 bp may be transformed into a 5000 × 300 × 4 vector through monomer encoding, with each column denoting a nucleotide and each row representing a feature. Similarly, the matrix dimensions with an equivalent window size for dimer encoding would be 5000 × 299 × 8. Figure 3 displays the 8-bit dimer encoding representation for all the 16 dinucleotides.

The transformation of a 300 bp sequence using dimer encoding is not being presented here comprehensively; rather, a smaller representation, the single dimer encoding of a 30 bp sequence of ‘CACACAAATAGTATACATCAAAAATGATTT’ has been encoded and shown in Table 1.

### 2.3. Model Training and Evaluation

**Figure 4:**
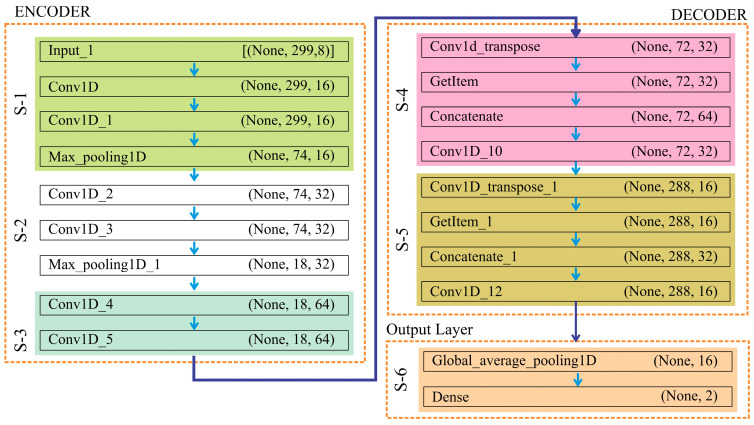
The architecture of the proposed classification model used to predict DNA methylation.

In this work, UNet employed the feature encoding technique to predict classification in an alphanumeric dataset. After their extraction, the pertinent features were divided into 70:20:10 as training, testing, and validation sets, respectively. In this experiment, an unidentified dataset of 1000 CG sites was used to test a neural net-based classification algorithm trained using 3500 methylation patterns of CGs, which was further validated on 500 CGs. The model’s architecture and parameters were carefully considered during creation to maximize the classification and prediction of DNA methylation patterns. Figure 4 describes the construction of this algorithm.

The encoder, decoder, and output layer are the three sub-components that form the complete architecture of UNet. The UNet architecture used here is specifically designed for a 1-dimensional alphanumeric dataset to stabilize and speed up the training process. It is a fully convolutional network composed of two main parts: the encoder (down-sampling path) and the decoder (up-sampling path). This architecture suits tasks requiring high spatial precision due to its ability to capture both low- and high-level features. The pseudocode (Appendix A) is described hereunder:

Encoder Block (Down-sampling Path): The encoder extracts hierarchical features from the input 1D sequence while progressively reducing its length. The process begins with an input of length 299 and 8 channels. It applies successive Conv1D layers with increasing filters $\left(16\to 32\to 64\right)$, each followed by MaxPooling1D to compress the sequence length $\left(299\to 74\to 18\right)$. This down-sampling captures increasingly abstract representations of the methylation patterns, while intermediate feature maps are stored for later skip connections in the decoder.

Decoder Block (Up-sampling Path): The decoder reconstructs the sequence length through Conv1DTranspose layers, integrating encoder features through skip connections to recover fine-grained details. At each stage, the sequence length expands $\left(18\to 72\to 288\right)$, while the number of filters decreases $\left(64\to 32\to 16\right)$. This progressive up-sampling restores the resolution of the sequence while retaining the hierarchical context learned in the encoder.

Output Layer: After the decoder reconstruction, a Global Average Pooling 1D (GAP) layer compresses the feature maps into a fixed-length vector, ensuring a compact representation and reducing the risk of overfitting. Finally, a fully connected dense layer with softmax activation outputs two probability scores corresponding to the methylated (M) and unmethylated (U) classes. The class with the higher probability is assigned as the predicted label.

The sklearn.metrics module prepares a confusion matrix to illustrate the model’s classification accuracy graphically and assists in compiling assessment metrics for the performance [17]. The number of target classes, “N”, is represented by a confusion matrix of N×N [18]. The matrix compares the actual target values with the machine learning model’s anticipated values.

The confusion matrix was used to calculate several standard evaluation metrics, including Sensitivity (SN), Specificity (SP), Accuracy (ACC), Matthews Correlation Coefficient (MCC), F-1 score, Area Under the Receiver Operating Characteristic (AUROC) Curve, and Area Under the Precision–Recall Curve (AUPRC) [19]. The following are the formulae for these metrics:

(2) $$\mathrm{SN}=\frac{\mathrm{TP}}{\mathrm{TP}+\mathrm{FN}}$$

(3) $$\mathrm{SP}=\frac{\mathrm{TN}}{\mathrm{TN}+\mathrm{FP}}$$

(4) $$\mathrm{ACC}=\frac{\mathrm{TP}+\mathrm{TN}}{\mathrm{TP}+\mathrm{TN}+\mathrm{FP}+\mathrm{FN}}$$

(5) $$\mathrm{MCC}=\frac{\mathrm{TP}\cdot \mathrm{TN}-\mathrm{FP}\cdot \mathrm{FN}}{\sqrt{\left(\mathrm{TP}+\mathrm{FP}\right)\left(\mathrm{TP}+\mathrm{FN}\right)\left(\mathrm{TN}+\mathrm{FP}\right)\left(\mathrm{TN}+\mathrm{FN}\right)}}$$

where TN and FN reflect the incidence of True Negatives (TN) and False Negatives (FN), respectively, whereas TP and FP show the number of True Positives (TP) and False Positives (FP). A thorough evaluation of a classifier is provided by the F-1 score, an evaluation statistic that considers both ACC and SN values. In addition, the precision score was used as a metric, which measures the ratio of accurately estimated positive class values to all expected positive class values.

The AUROC curve and the AUPRC were computed on the basis of the Receiver Operating Characteristic (ROC) curve and the Precision–Recall (PR) curve, respectively, to evaluate the performance of different models comprehensively [20,21]. Graphs of the Validation Accuracy (VA) and Validation Loss (VL) curves were used for visualization analysis [22]. All visualizations were built using Matplotlib (version 3.7.0), a complete Python (version 3.13) framework for interactive visualizations [23]. The final results, which comprised several performance metrics, were then carefully organized into a pandas DataFrame to derive greater understanding.

### 2.4. Validation of Promoter-Region Genes

**Figure 5:**
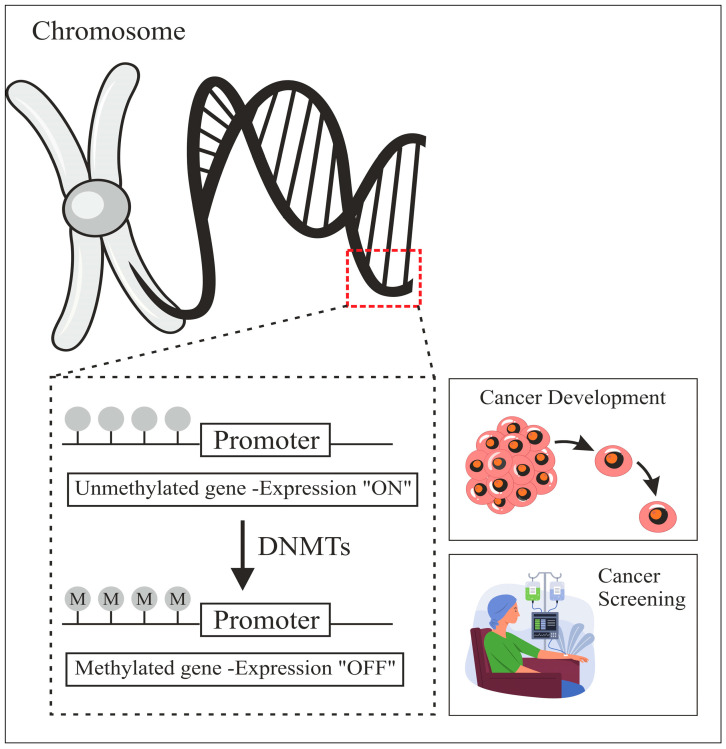
Influence of DNA methylation in regulating gene expression of the promoter region.

The proposed model was validated using methylation data from the promoter regions of five genes: *miR-100*, *miR-138*, *miR-484*, *hTERT*, and *ERVH48-1*. Previous studies have established the association of promoter methylation in these genes with cervical cancer cell lines (as illustrated in Figure 5) [24,25,26,27,28]. As many as 128 CG sites in the promoter regions of these genes were considered for methylation value determination in HeLa cells based on experimentally obtained data from prior studies [24,25,26,27,28].

The methylation data was converted into a binary dataset using a previously defined β value and was subsequently cleaned to remove outliers, resulting in a final dataset of 87 CG sites. For further analysis, methylation patterns within a 300 bp window centered around each CG site were extracted using bisulfite-sequenced data from the NCBI human genomic assembly. The final dataset was used to evaluate the proposed model, demonstrating its robustness.

### 2.5. State-of-the-Art Methods

In this study, CNN and MobileNet were employed as baseline state-of-the-art methods for methylation prediction and compared against the proposed UNet architecture. While all three models are convolutional in nature, their designs differ substantially. As a result, the most effective model for precise predictions may be determined by comparing the differences in architectural optimization, hyperparameter tuning, and efficiency.

#### 2.5.1. Proposed Convolutional Neural Network

Keras and TensorFlow were used to define the proposed CNN model [29]. The model consists of sequential convolutional layers with trainable kernels, non-linear activation functions, and pooling operations. Convolutional operations were defined as demonstrated below in Equation (6):

(6) $$F\left(i\right)=\left(I\times K\right)\left(i\right)=\sum_{m=0}^{k-1}I\left(i+m\right)K\left(m\right)$$

where “I” stands for the input matrix, “K” for the 1-D filter of length “k”, and “F” for the 1-D feature map’s output [30,31,32,33]. “I×K” is used to symbolize the convolutional layer. The architecture extracts hierarchical spatial representations that serve as a baseline for comparison.

#### 2.5.2. Proposed Mobile Net

The second baseline, MobileNet, was designed using depth-wise separable convolutions, which decouple standard convolution into channel-wise filtering and point-wise convolution [34]. The architecture integrates bottleneck layers, linear bottlenecks, inverted residual connections, and squeeze-and-excitation (SE) blocks, enabling compact yet expressive feature learning suitable for the methylation prediction task.

Performance comparison among CNN, MobileNet, and the proposed UNet was subsequently carried out to evaluate predictive effectiveness in methylation status classification.

## 3. Results and Discussion

Predictions made through AI models were used extensively for several normal and malignant cell lines, as listed in Table 2. Zeng et al. [35] presented CpGenie in 2017, which utilized a Convolutional Neural Network (CNN) to examine the impact of sequence heterogeneity on CpG methylation, achieving an Accuracy (ACC) of over 90.00%. Tian et al. [36] proposed MRCNN, which used the correlation between DNA sequence patterns and their methylation state to predict the methylation of the CpG site at an accurate single-base resolution. To obtain the expected value, the model used completely linked layers, convolution, pooling, and one-hot encoding. Using a continuous loss function, it achieved an apparent regression of methylation with 93.20% accuracy. The 2D convolution technique used supplementary DNA sequence characteristics to enhance methylation prediction. Nonetheless, expected outcomes remained constant, provided the sequence was defined. In 2019, Fu et al. [37] used a novel CNN and Feedforward Neural Network (FFNN) combination that combined DNA sequence data with MeDIP-seq and histone modification data to study the prediction of methylation states. The model outperformed conventional techniques and demonstrated a significant increase in predicting DNA methylation states with an excellent Area Under Curve (AUC) of 97.70. According to the findings of this study, ACC of DNA methylation state prediction improved significantly by combining epigenomic and DNA sequence data. Another combination of Recurrent Neural Network (RNN) with CNN models was presented by Wu et al. [9]. This fusion Deep Learning (DL) method could predict the methylation status of DNA with an ACC of 84.90%. RNNs performed well when extracting sequential attributes, but Wu et al. had issues finding correlations between extremely distant elements. The recent study conducted by Mallik et al. [38] reported the prediction of DNA methylation in cervical cancer through the use of FFNN with an accuracy of 90.69%. However, using this model usually resulted in loss of neighborhood information, which could have been crucial in studying DNA patterns, as neighborhood sequences highly influenced Cytosine–Guanine (CG) methylation. Apart from this, Ma et al. [39] used Cox Regression analysis to identify 34,389 differentially methylated CpG sites and built a model based on four CpG sites, demonstrating an AUC of 83.30.

A review of the existing literature led to the fact that analyzing the proposed model on the basis of sample size and window size can provide crucial insight into the mechanism of DNA methylation in multiple cancer cell lines. The proposed model was also used to predict methylation in the promoter-region genes associated with cervical cancer to validate its reliability. It outperformed the existing state-of-the-art algorithms, *viz*., CNN and MobileNet, with 91.60% accuracy in predicting this epigenetic modification in cervical cancer, as illustrated in Table 3 by several evaluation metrics for various sample sizes of CGs. A detailed interpretation of extensive experimentation is provided in the following subsections:

### 3.1. Comparative Assessment Showing the Influence of All Three Models on the HeLa Cell Line with Different Sample Sizes Using Varied Encoding Techniques and Window Sizes on the Basis of Evaluation Metrics

The comparative analysis of CNN, MobileNet, and UNet across varying encoding schemes, window sizes, and sample sizes showed distinct performance patterns that underscore the strengths and limitations of each architecture. Overall, UNet consistently outperformed CNN and MobileNet in most experimental settings, particularly in terms of ACC, SP, and F-1 score, suggesting its superior capacity to capture spatial dependencies in encoded sequences. For the monomer encoding, UNet exhibited robust performance across all window sizes, maintaining ACC above 80% for most cases except at the 300 bp window setting, where CNN surpassed it. Interestingly, CNN demonstrated relatively balanced SN and SP in larger window sizes, particularly at 300, where it achieved its highest ACC of around 83%. MobileNet, however, consistently performed poorly compared to other models, with performance metrics such as MCC and F-1 score remaining comparatively lower, highlighting its limited suitability for this classification task.

In the dimer encoding scheme, UNet surpassed other models at a 300 bp window size and a smaller sample (5000), achieving ACC of 91.60%, MCC of 83.72%, and F-1 score of 91.30%. CNN followed closely, performing particularly well in terms of SN, indicating its ability to minimise FN. This trend reinforces that dimer encoding, by encapsulating higher-order nucleotide information, enhances discriminative capacity when paired with deeper models like UNet. MobileNet’s performance improved modestly under dimer encoding but lagged behind both UNet and CNN. Across sample sizes, a general decline in performance with increasing data volume was observed, particularly for UNet under monomer encoding. This may be attributed to model overfitting on smaller datasets, where patterns are more localized, whereas larger datasets cause complexity and noise. Conversely, CNN appeared to benefit from larger sample sizes at higher window dimensions, suggesting its resilience in capturing broader contextual patterns.

Overall, the results highlight that UNet, coupled with dimer encoding and moderate window sizes (200–300), is the most effective strategy for this classification task, balancing SN and SP while maintaining high precision. CNN emerged as a competitive alternative at larger window sizes, while MobileNet consistently underperformed, indicating limited adaptability in this experimental domain.

### 3.2. Assessing the Influence of All Three Selected Models on Different Cell Lines Using Various Sample Sizes

The performance of UNet with dimer encoding and a window size of 300 was better than the other two models used to predict methylation in the cervical cell line. Thus, this particular window size and encoding were then used to assess its performance by validating it on the three different sample sizes for HepG2, a liver cancer cell line. A comprehensive evaluation of all the selected models with this combination of encoding and window size is made in the following subsections with the help of confusion matrices, learning curves, and visualization aids.

#### 3.2.1. Assessing the Performance of the Proposed Model in Comparison to CNN and MobileNet on 5000 CG Sites of the HepG2 Cell Line

**Figure 6:**
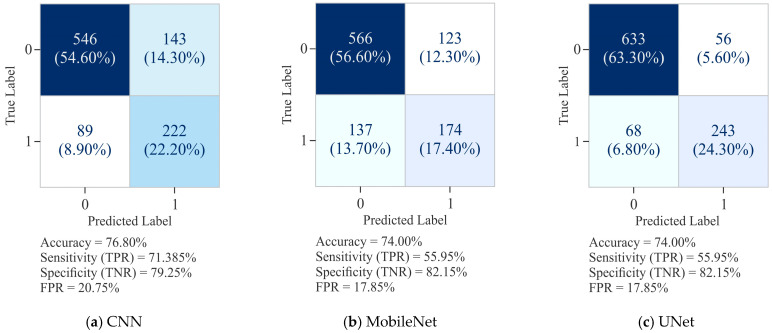
Confusion matrix for 5000 samples using the Dimer Encoding Technique with a window size of 300 on the HepG2 Cell Line.

It can be observed from Figure 6a that the CNN model attained an overall ACC of 76.80%, with SP (TNR) of 79.25% and SN (TPR) of 71.38%. While CNN demonstrated a reasonably balanced performance, it exhibited a tendency to misclassify positive cases, as reflected in the greater number of FN. In comparison, MobileNet recorded a slightly lower ACC of 74.00%. Its specificity improved to 82.15%, thereby reducing the FPR to 17.85%, but this improvement came with lower SN, which dropped to 55.95%. This indicates that MobileNet was more conservative in predicting positive cases, leading to a considerable increase in missed detections (FN = 137). On the other hand, UNet demonstrated better performance across all the evaluation metrics with an ACC of 87.60%, SN of 78.14%, and SP of 91.87%. Notably, its FPR was the lowest at 8.13%, while simultaneously reducing FN to 68, thereby achieving a better trade-off between positive and negative classification. These results highlight that UNet outperformed both by maintaining high SP and substantially improving SN, making it the most robust model for reliable classification in this dataset.

**Figure 7:**
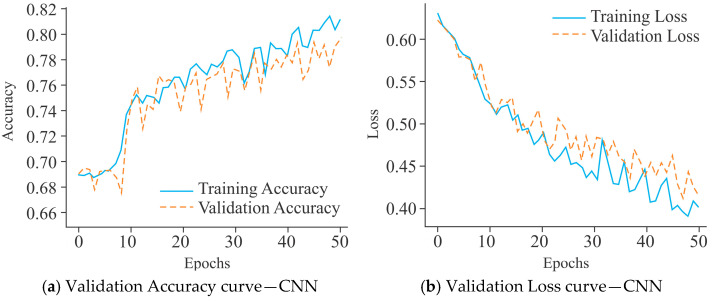

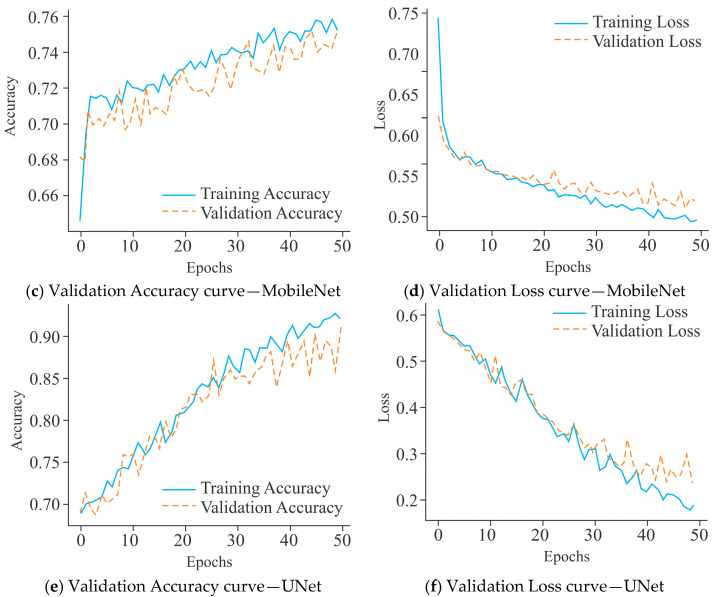
Learning curves of various models using the Dimer Encoding Technique on the HepG2 Cell Line with a window size of 300 using 5000 samples, represented by the VA curve and the VL curve.

The CNN model, as shown in Figure 7a, demonstrated steady improvement in training accuracy, reaching approximately 82% after 50 epochs. Its validation accuracy, however, stabilized around 76%, with noticeable oscillations, indicating moderate overfitting and limited generalization capacity. In contrast, MobileNet (Figure 7c) converged more slowly, with training accuracy peaking near 76%, while validation accuracy remained lower at approximately 72%, accompanied by larger fluctuations. This trend suggests that MobileNet struggled to capture the underlying data distribution effectively, leading to weaker performance in validation compared to CNN. On the other hand, UNet (Figure 7e) exhibited a markedly superior performance profile, with training accuracy rising to over 90% and validation accuracy stabilizing above 87% with fewer fluctuations. This indicates better convergence behavior and more generalization as compared to CNN and MobileNet.

The CNN model presented in Figure 7b exhibited a progressive decline in training loss, which stabilized near 0.40 after 50 epochs. However, the validation loss remained consistently higher and displayed significant fluctuations beyond the 20th epoch, which is indicative of moderate overfitting and limited generalization. MobileNet (Figure 7d) also showed a marked reduction in training loss, converging around 0.49, while the validation loss stabilized near 0.54 with recurrent oscillations. The divergence between training and validation loss underscores that MobileNet has a greater tendency to overfit, with weaker generalization compared to CNN. In contrast, UNet (Figure 7f) demonstrated a more favorable loss profile, with training loss decreasing steadily to below 0.20 by the 50th epoch and validation loss stabilizing around 0.30–0.35. Although fluctuations in validation loss were present, they were less pronounced and remained within a narrower margin compared to CNN and MobileNet. These observations suggest that UNet achieved the most balanced optimization, offering reduced overfitting and enhanced generalization capability across the models evaluated.

**Figure 8:**
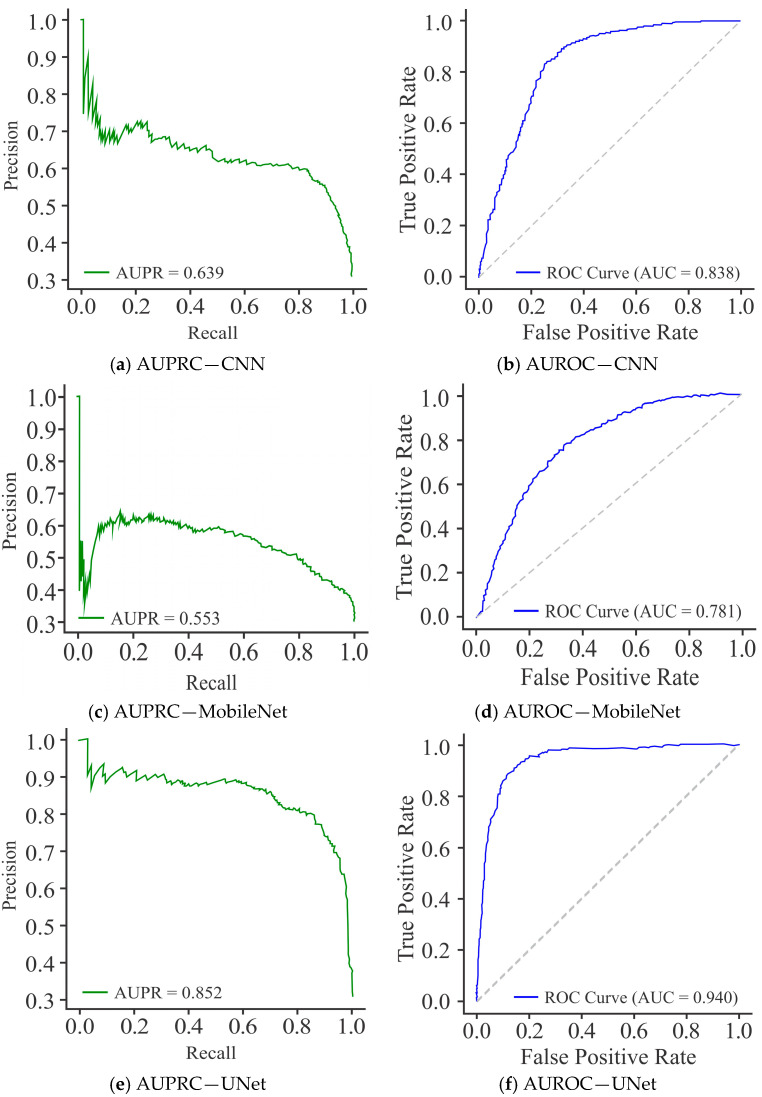
Visualization curves of various models derived through the Dimer Encoding Technique on the HepG2 Cell Line with a window size of 300 using 5000 samples, represented by the AUPRC and AUROC curves.

The CNN model, as shown in Figure 8a, demonstrated a moderate level of precision levels, but the curve fluctuated substantially across recall values, yielding an average precision (AUPR) of 0.637. This variability reflects the model’s inconsistency in maintaining precision at different classification thresholds, suggesting decision imbalance. In comparison to the CNN model, MobileNet (Figure 8c) showed a smoother but overall weaker PR curve, with an AUPR of 0.553, reflecting reduced discriminative capability. While it was able to maintain reasonable recall, the decline in precision with increasing recall values indicates a stronger bias toward recall, limiting its practical reliability. In comparison, UNet (Figure 8e) recorded better performance, with a high and stable PR curve and an AUPR of 0.851. Its precision remained consistently elevated across a wide range of recall values, indicating robust trade-off management and strong generalization capacity.

The AUROC curve, shown in Figure 8b, reflects that the CNN model attained an AUROC of 0.837, indicating good discriminative performance in distinguishing methylated from unmethylated sites. The curve follows a stable upward trajectory, maintaining a reasonable balance between SN (TPR) and SP, though sufficient scope exists for further enhancement. In comparison, MobileNet (Figure 8d) yielded a lower AUROC of 0.781, signifying reduced discriminative ability relative to CNN. While its ROC curve is able to maintain the expected rising shape, the reduced separation from the diagonal line reflects the model’s weak performance, particularly at higher FPR. On the other hand, UNet (Figure 8f) achieved the highest AUROC of 0.939, demonstrating its superior classification capability. Its ROC curve appears closer to the top-left corner, representing an optimal trade-off between SN and SP. On the basis of these results, it can be said that although CNN provided a solid baseline and MobileNet performed less effectively, UNet displayed the greatest discriminative power with minimal FP, making it the most reliable model for DNA methylation site classification.

#### 3.2.2. Performance Assessment of the Proposed Model in Comparison to CNN and MobileNet on 10,000 CG Sites of the HepG2 Cell Line

**Figure 9:**
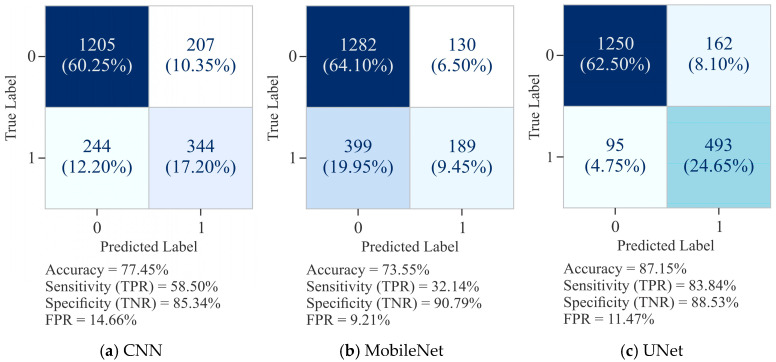
Confusion matrix for 10,000 samples using the Dimer Encoding Technique with a window size of 300 on the HepG2 Cell Line.

The confusion matrix for CNN, as shown in Figure 9a, registered an overall ACC of 77.45%, with SP (TNR) of 85.34% and SN (TPR) of 58.50%. This shows that CNN was effective in minimizing FP (FPR = 14.66%), but its relatively low SN suggests difficulty in identifying positive cases, as evidenced by a higher proportion of FN, being 244. In comparison, MobileNet, as presented in Figure 9b, recorded a slightly lower ACC of 73.55%. While its SP increased to 90.79% with a reduced FPR of 9.21%, its SN dropped to 32.14%, reflecting substantial misclassification of positive instances (FN = 399). This result suggests that the performance of MobileNet was as good as that of CNN in detecting TP. On the other hand, UNet, as depicted in Figure 9c, clearly outperformed both CNN and MobileNet by maintaining the optimal balance between SN and SP. It recorded an ACC of 87.15%, with SN of 83.84% and SP of 88.53%, alongside a comparatively low FPR of 11.47%. UNet’s ability to minimize FP to 162 and FN to 95 demonstrates its robustness in effectively capturing accurate signals while avoiding overprediction of positives.

**Figure 10:**
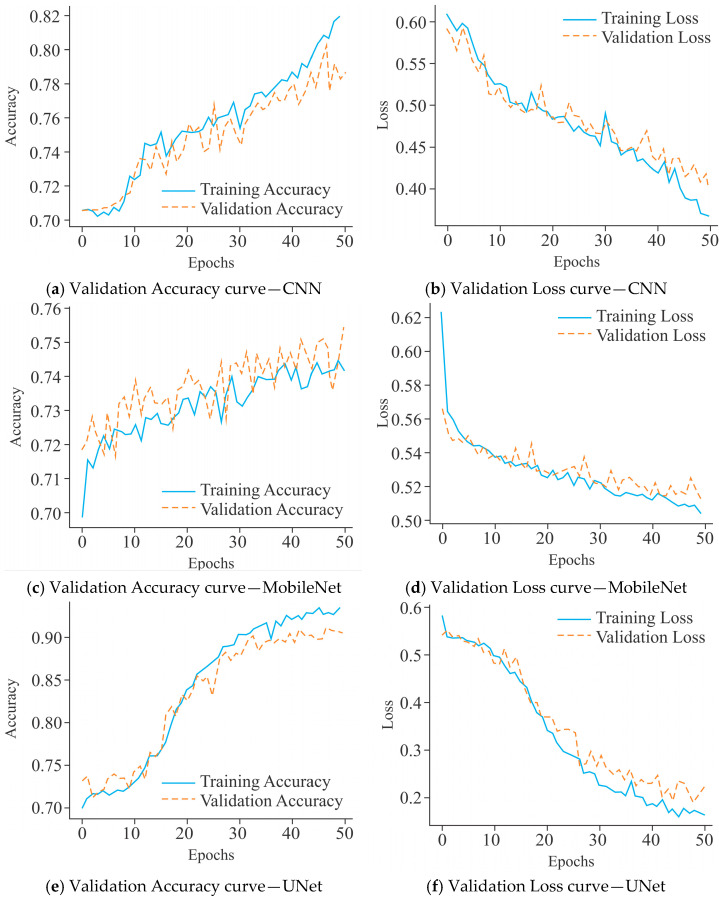
Learning curves of various models using the Dimer Encoding Technique on the HepG2 Cell Line with a window size of 300 using 10,000 samples, represented by the VA curve and VL curve.

The VA and VL curves for CNN, MobileNet, and UNet, as shown in Figure 10, highlight their learning dynamics and generalization capabilities when predicting DNA methylation on 10,000 CGs. The CNN model (Figure 10a) exhibited a steady improvement in training accuracy, reaching approximately 82% after 50 epochs. However, the validation accuracy remained lower, around 75%, with noticeable fluctuations, indicating partial overfitting and limited generalization capacity. In comparison, MobileNet (Figure 10c) displayed a relatively constrained training progression, with accuracy stabilizing near 74%, while validation accuracy fluctuates heavily, occasionally surpassing the training curve but lacking stability. This erratic pattern suggests inconsistent learning behavior and weak generalization capability. In comparison, UNet (Figure 10e) demonstrated the most favorable trend, with training accuracy steadily rising above 92% and validation accuracy stabilizing at approximately 86%. The smaller gap between training and validation accuracy, along with reduced oscillations, indicates UNet’s greater generalization and robustness.

Further, the CNN model (Figure 10b) demonstrated a steady decline in training loss, reaching approximately 0.37 by the end of 50 epochs. However, the validation loss remained consistently higher, fluctuating between 0.46 and 0.50, indicating moderate overfitting as the model learned the training distribution more effectively. MobileNet (Figure 10d) exhibited a slower but stable decline in training loss, converging near 0.51. Its validation loss, however, persisted above 0.52 with oscillations throughout the epochs, suggesting that MobileNet struggled to minimize error on validation data and reflects weaker generalization ability. On the other hand, UNet (Figure 10f) showed a pronounced reduction in training loss, reaching below 0.15 by epoch 50, accompanied by relatively lower and more stable validation loss, stabilizing between 0.35 and 0.40. While minor fluctuations existed, the validation loss of UNet did not diverge drastically from the training curve, indicating greater generalization. Collectively, these results emphasize that UNet offers the most reliable convergence, balancing training and validation performance more effectively than CNN and MobileNet.

**Figure 11:**
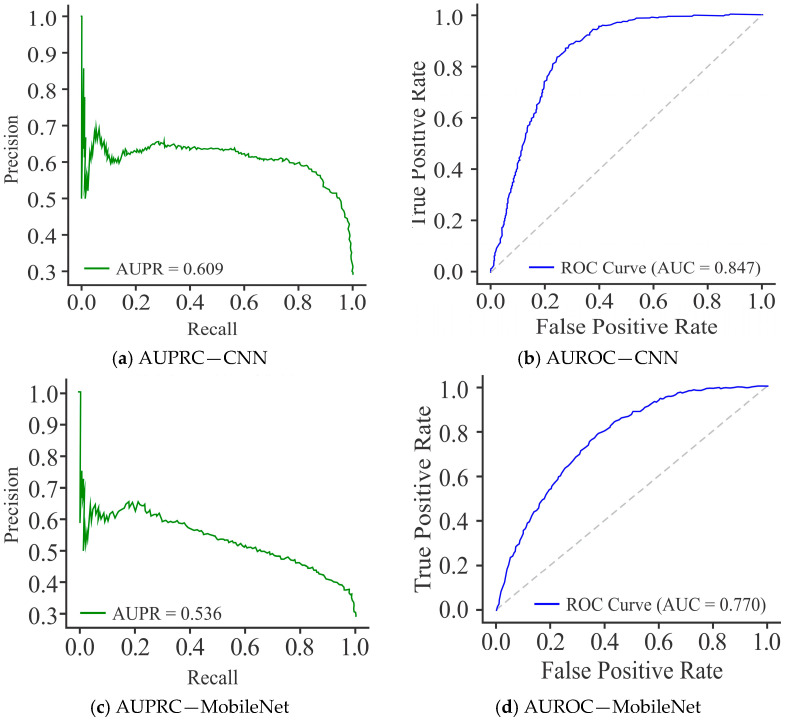

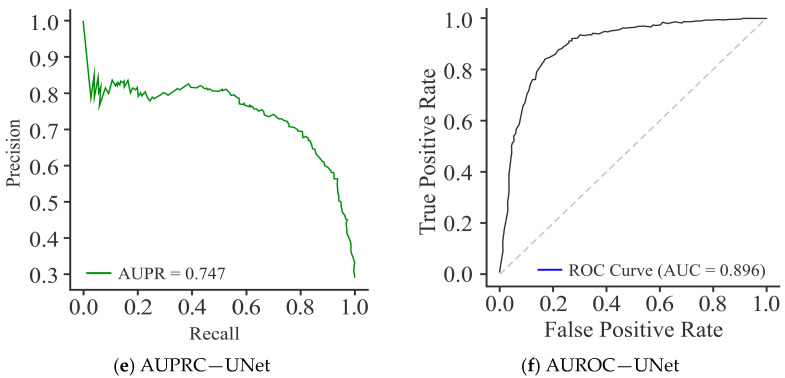
Visualization curves of various models using the Dimer Encoding Technique on the HepG2 Cell Line with a window size of 300 using 10,000 samples, represented by AUPRC and AUROC curves.

The PR curves for the CNN model, as shown in Figure 11a, achieved an AUPRC score of 0.609, reflecting moderate performance. However, precision dropped sharply at higher recall values, indicating that CNN struggled to maintain consistent precision across thresholds. The performance of MobileNet (Figure 11c) was not as good as that of the CNN model, with the lowest AUPRC of 0.536, and consistently reduced precision across the recall spectrum, highlighting its limited discriminative ability and poor balance between precision and recall. In comparison, UNet (Figure 11e) demonstrated a clear advantage by achieving the highest AUPRC of 0.746. Its curve remained relatively smooth, with precision values consistently higher over a broad recall range, especially in the mid-to-high recall regions. This stability indicates that UNet established a better trade-off between precision and recall than CNN and MobileNet.

As shown in Figure 11b, the CNN model attained an AUROC of 0.847, indicating good discriminative capacity in separating methylated from unmethylated sites. Its curve demonstrates a stable trade-off between sensitivity and specificity. MobileNet (Figure 11d) yielded the lowest AUROC of 0.770, reflecting weaker discriminative ability and closer alignment to the diagonal, particularly at higher FPR. This underlines its limited suitability for robust methylation site classification. In contrast, the performance of UNet (Figure 11f) was the best, with an AUROC of 0.896, and its curve lay closer to the top-left corner, demonstrating an optimal balance between TP and FPR. These results establish that while CNN offered a strong baseline and MobileNet performed sub-optimally, UNet provided the highest discriminative ACC and generalization, making it the most effective model for this classification task.

#### 3.2.3. Assessing the Performance of the Proposed Model with CNN and MobileNet on 20,000 CG Sites of the HepG2 Cell Line

**Figure 12:**
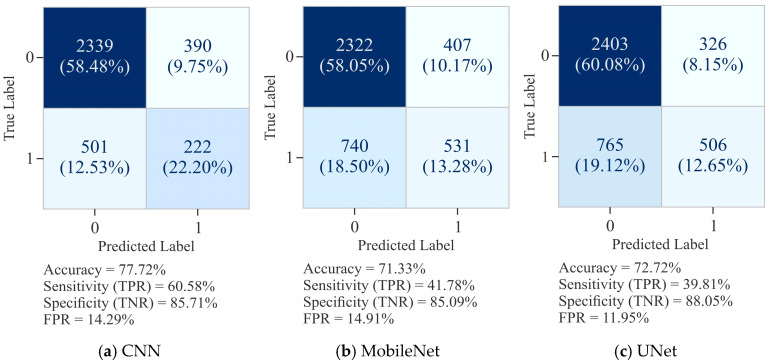
Confusion matrix for 20,000 samples using the Dimer Encoding Technique with a window size of 300 on the HepG2 Cell Line.

The confusion matrices for CNN, MobileNet, and UNet with 20,000 samples, as shown in Figure 12, present comparative results in predicting DNA methylation. The CNN model (Figure 12a) exhibited the highest overall ACC of 77.72%, supported by SP (TNR) of 85.71% and SN (TPR) of 60.58%. While CNN effectively limited FP (FPR = 14.29%), its relatively lower SN reflects a tendency to miss positive cases (FN = 501). MobileNet (Figure 12b) recorded an ACC of only 71.33%, with SP remaining comparable at 85.09% but SN dropped significantly to 41.78%, reflecting its difficulty in correctly identifying methylated sites (FN = 740). Although the FPR was similar to that of CNN (FPR = 14.91%), its lower SN limited the scope of this model for the task.

The results produced by UNet (Figure 12c) were balanced but not superior to those of CNN. The ACC was 72.72%, SP 88.05%, and FPR 11.95%. However, its SN was only 39.81%, indicating that while UNet was able to effectively minimize FP, it struggled to capture positive cases (FN = 759). The overall results confirmed that CNN maintained the best trade-off between SN and SP at 20,000 samples, while UNet leaned more toward conservative predictions with higher SP but lower SN. In contrast, MobileNet underperformed with respect to SN and ACC, making it the least effective model for reliable DNA methylation classification in this setting.

**Figure 13:**
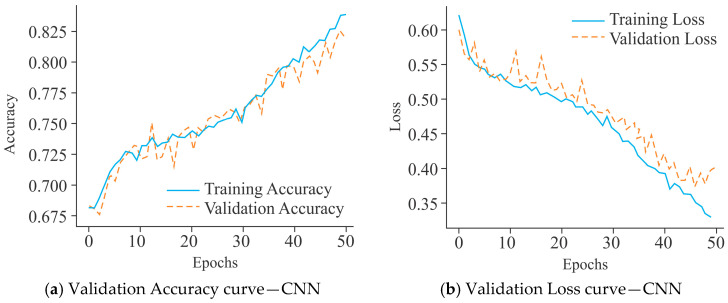

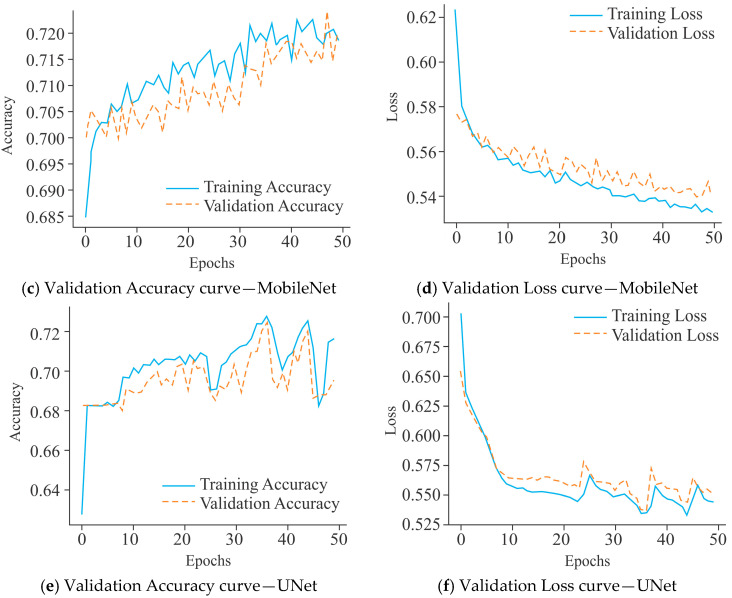
Learning curves of various models using the Dimer Encoding Technique on the HepG2 Cell Line with a window size of 300 using 20,000 samples, represented by VA curves and VL curves.

The VA curves for the CNN model, as shown in Figure 13a, demonstrated a constant upward trajectory, with training accuracy of 82% and validation accuracy stabilizing around 77% after 50 epochs. Although a small gap existed between training and validation accuracy, the fluctuations remained moderate, suggesting limited overfitting and acceptable generalization performance. In comparison, the performance of MobileNet (Figure 13c) was not as good as that of the CNN model. Its training accuracy was about 72%, while validation accuracy fluctuated heavily around 70–71%, occasionally dipping lower. The instability of the validation curve reduced the generalization of the model and indicated a stronger tendency toward overfitting despite lower training accuracy. On the other hand, UNet (Figure 13e) maintained a relatively better alignment between training and validation accuracy, both stabilizing around 71–72%. Although fluctuations were visible, they remained less pronounced compared to MobileNet, reflecting its greater generalization. Overall, these findings reflect that CNN outperformed MobileNet and UNet models in terms of accuracy stability, while UNet maintained a comparatively balanced generalization profile, making it more reliable than MobileNet under the same experimental conditions.

The training and VL curves for the CNN model, as shown in Figure 13b, demonstrated a consistent reduction in training loss, converging to approximately 0.33 after 50 epochs. However, its validation loss remained comparatively higher with recurrent fluctuations, indicating moderate overfitting and less generalization. MobileNet (Figure 13d) showed a meagre decline in training loss, stabilizing near 0.53, while its validation loss persisted above 0.55 with frequent oscillations. This pattern reflects limited capacity of the model to minimize error on unseen data, with stronger evidence of overfitting in comparison to the CNN model. In comparison, the UNet model (Figure 13f) displayed a more stable loss behavior, with training loss steadily declining to around 0.54 and validation loss closely following a parallel trajectory near 0.55. Although minor fluctuations persisted, the reduced gap between training and validation losses reflects better convergence and greater generalization compared to the CNN and MobileNet models.

**Figure 14:**
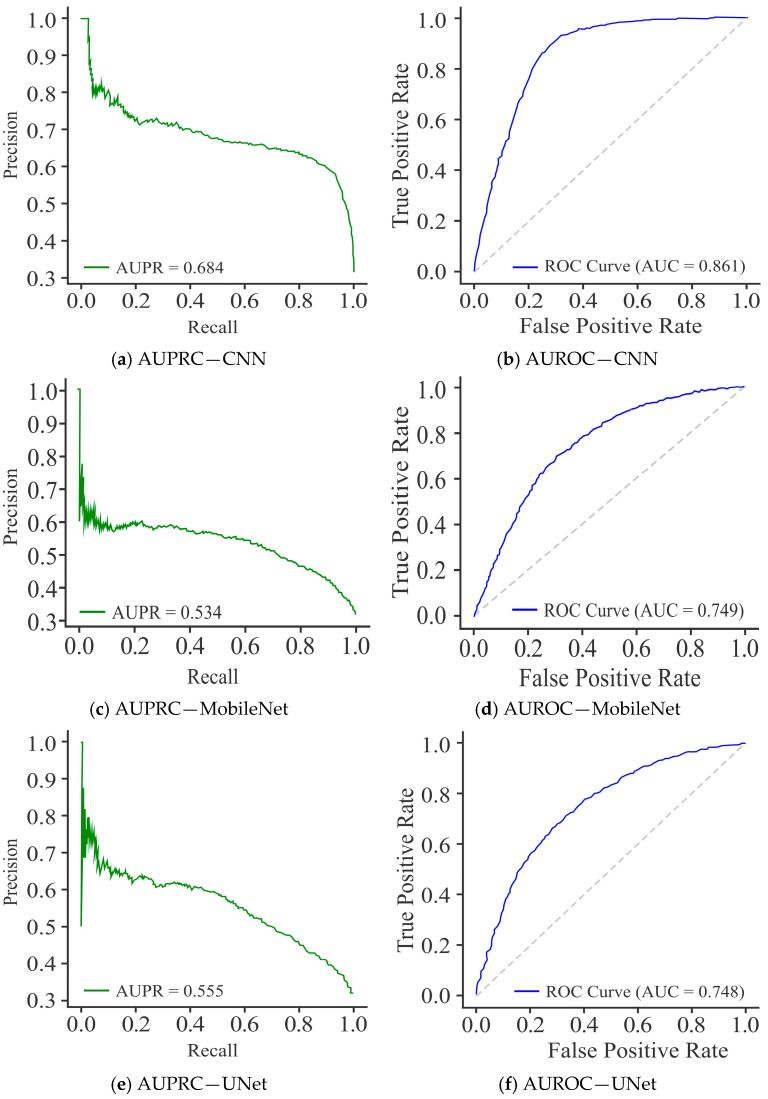
Visualization curves of various models using the Dimer Encoding Technique on the HepG2 Cell Line with a window size of 300 using 20,000 samples, represented by AUPRC and AUROC curves.

Figure 14a evidently shows that the CNN model achieved an AUPR of 0.684, reflecting moderate performance with reasonable precision maintained over the mid-recall range, although fluctuations were evident at lower recall values. This indicates some instability in precision when the recall is small, limiting its reliability across thresholds. The performance of the MobileNet model (Figure 14c) was the worst, with the lowest AUPRC of 0.534 and a consistently declining precision curve across recall values, highlighting poor discriminative capacity and lower generalization in comparison to the CNN and UNet models. The UNet model (Figure 14e), with an AUPRC of 0.555, achieved slightly better performance than the MobileNet model, but it performed considerably less than the CNN model. Its curve showed a smoother trend than MobileNet but failed to sustain high precision at increasing recall levels.

The ROC curves for all three selected models were used for a comparative evaluation of their classification performance on the HepG2 cell line. The CNN model, as shown in Figure 14b, recorded an AUROC of 0.861, indicating its firm discriminative ability with a good balance between SN and SP. The performance of the MobileNet model, as shown in Figure 14d, was the worst, with an AUROC of 0.749, reflecting its limited capacity to maintain high TPR at low FPR. The UNet model, as shown in Figure 14f, registered the highest AUROC of 0.896, with its curve lying closest to the top-left corner, demonstrating superior classification performance and better generalization. The overall results show that the performance of CNN model was quite reliable, while the MobileNet model failed to perform satisfactorily. However, UNet emerged as the most effective model for accurate methylation site prediction.

### 3.3. Validation Using CGs Present in the Promoter Region

As mentioned earlier, the methylation status of the promoter regions of five genes, *viz.*, *miR-100*, *miR-138*, *miR-484*, *hTERT*, and *ERVH48-1*, was utilized to validate the proposed framework. These genes exhibited distinct methylation patterns in their promoter regions, encompassing 87 CG sites around the TSS. These sites were classified as methylated or unmethylated on the basis of their association with cervical cancer, as reported in earlier studies. Thus, by obtaining an ACC of 73.00% in the promoter regions of the genes, the proposed model was validated on these CG locations to prove its robustness.

**Figure 15:**
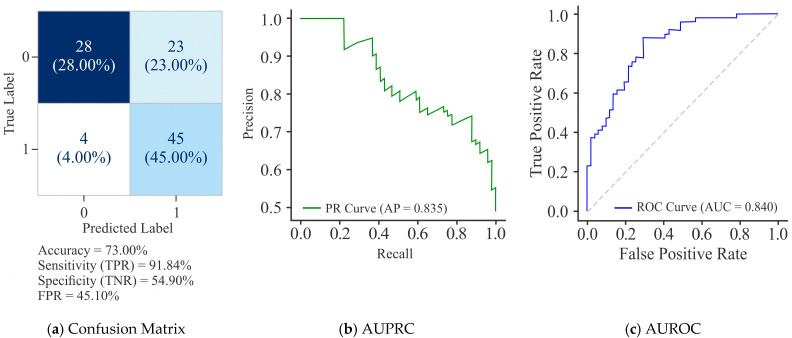
Performance of UNet in predicting DNA methylation on promoter-region genes linked with cervical cancer.

It can be observed from Figure 15 that the UNet model performed reasonably well, as it efficiently detected methylation of promoter-region genes. It correctly identified 86.27% of actual methylation in genes of promoter regions. This is particularly important in cancer research, where missing TP could mean overlooking critical methylation levels in certain gene regions. Of 51 CGs, 44 were accurately identified, as shown in Figure 15a. This highlights the model’s reliability in pinpointing these sites. With a balanced F-1 score, the model efficiently balanced precision and recall, making it the most reliable choice for applications requiring sensitivity and reliability. The AUPRC score of 0.835 suggests that the model maintained substantial precision while achieving high recall values (Figure 15b). The AUROC score of 0.839 signifies excellent overall performance of the model across various thresholds (Figure 15c). These results suggest that the model can confidently serve as a reliable tool in the initial stages of identifying methylation in cervical cancer-associated gene promoter regions, paving the way for further refinement and usage in critical research and diagnostics.

## 4. Conclusions, Limitations, and Future Work

This study demonstrated that combining a UNet architecture with dimer encoding effectively classifies DNA methylation patterns in cervical cancer, achieving an Accuracy of 91.60%, Sensitivity of 96.71%, Specificity of 87.32%, and an AUROC of 96.53. Systematic evaluation across sample sizes and sequence window lengths identified 5000 CG sites with a 300 bp window as the optimal configuration. Validation on five cervical cancer–associated gene promoter regions confirmed the model’s robustness, correctly identifying 86.27% of methylated sites. These results are, without any doubt, better than those produced through the application of prior encoding and normalization approaches, underscoring the value of deep learning-based feature extraction for epigenetic biomarker discovery. However, the framework’s generalizability requires further validation on larger and more diverse clinical datasets. Future work can be undertaken on the model by extending it to real-world cohorts and integrating additional omics data (epigenomics and genomics) to enhance its applicability in clinical diagnostics.

## Figures and Tables

**Table 1:** Dimer encoding representation for a 30 bp sequence—“CACACAAATAGTATACATCAAAAATGATTT”.

	Base Sequence	A	C	G	T	A	C	G	T
**1.**	**CA**	0	1	0	0	1	0	0	0
**2.**	**AC**	1	0	0	0	0	1	0	0
**3.**	**CA**	0	1	0	0	1	0	0	0
**4.**	**AC**	1	0	0	0	0	1	0	0
**5.**	**CA**	0	1	0	0	1	0	0	0
**6.**	**AA**	1	0	0	0	1	0	0	0
**7.**	**AA**	1	0	0	0	1	0	0	0
**8.**	**AT**	1	0	0	0	0	0	0	1
**9.**	**TA**	0	0	0	1	1	0	0	0
**10.**	**AG**	1	0	0	0	0	0	1	0
**11.**	**GT**	0	0	1	0	0	0	0	1
**12.**	**TA**	0	0	0	1	1	0	0	0
**13.**	**AT**	1	0	0	0	0	0	0	1
**14.**	**TA**	0	0	0	1	1	0	0	0
**15.**	**AC**	1	0	0	0	0	1	0	0
**16.**	**CA**	0	1	0	0	1	0	0	0
**17.**	**AT**	1	0	0	0	0	0	0	1
**18.**	**TC**	0	0	0	1	0	1	0	0
**19.**	**CA**	0	1	0	0	1	0	0	0
**20.**	**AA**	1	0	0	0	1	0	0	0
**21.**	**AA**	1	0	0	0	1	0	0	0
**22.**	**AA**	1	0	0	0	1	0	0	0
**23.**	**AA**	1	0	0	0	1	0	0	0
**24.**	**AT**	1	0	0	0	0	0	0	1
**25.**	**TG**	0	0	0	1	0	0	1	0
**26.**	**GA**	0	0	1	0	1	0	0	0
**27.**	**AT**	1	0	0	0	0	0	0	1
**28.**	**TT**	0	0	0	1	0	0	0	1
**29.**	**TT**	0	0	0	1	0	0	0	1

**Table 2:** Performance comparison between the proposed model and methods previously used for predicting DNA methylation in cancer cells.

S. No.	Author	Dataset	Pre-Processing	Model	ACC%	SN%	SP%	MCC%	AUROC
1.	Wu et al., 2022 [9]	GEO, GSE152204	CNN + RNN + One-hot encoding	ResNet	84.90	-	-	-	-
2.	Ma et al., 2022 [39]	miRNA + mRNA datasets for cervical cancer	Functional Normalization	Cox Proportional Hazard Regression Analysis model	-	-	-	-	83.30
3.	Mallik et al., 2020 [38]	Uterine Cervical cancer dataset from NCBI	Voom Normalization and Limma	FFNN	90.69	73.97	97.63	78.23	85.80
4.	Fu et al., 2019 [37]	Cell lines: GM12878 and K562	Convolutional layers + One-hot encoding		-	-	-	-	97.70
5.	Tian et al., 2022 [36]	Non-cancerous: H1-ESC; Cancerous: white matter of brain, lung tissue and colon tissue	One-hot encoding	CNN	>93.20	>95.00	85.00	-	96.00
6.	Zeng et al., 2017 [35]	50 human cancer cell lines			90.00	-	-	-	85.40
**7.**	**Proposed method**	**HeLa, HepG2**	**Dimer encoding**	**UNet**	**91.60**	**96.71**	**87.32**	**83.72**	**96.53**

(ACC: Accuracy; SN: Sensitivity; SP: Specificity; MCC: Matthews Correlation Coefficient; AUROC: Area Under Receiver Operating Characteristic curve).

**Table 3:** Comparative performance of CNN, MobileNet, and UNet in terms of evaluation metrics on the basis of character encoding for the three different window sizes for sample sizes 5000, 10,000, and 20,000.

S. No.	Window Size	Encoding	Sample Size	Model	ACC%	SN%	SP%	MCC%	Precision Score	F-1 Score
1.	100	Monomer	5000	UNet	89.40	86.40	91.91	78.62	89.95	88.14
2.				CNN	76.00	66.45	84.01	51.52	77.69	71.63
3.				MobileNet	69.80	61.84	76.47	38.80	68.78	65.13
4.			10,000	UNet	83.15	84.13	82.27	66.31	80.96	82.51
5.				CNN	71.05	70.37	71.66	41.99	68.98	69.67
6.				MobileNet	70.30	65.35	74.45	39.96	68.19	66.74
7.			20,000	UNet	81.77	80.92	82.60	63.54	81.88	81.40
8.				CNN	74.68	79.81	69.69	49.71	71.89	75.64
9.				MobileNet	68.00	46.49	86.03	35.77	73.61	56.99
10.	100	Dimer	5000	UNet	87.30	84.65	89.52	74.37	87.13	85.87
11.				CNN	76.20	76.97	75.55	52.35	72.52	74.68
12.				MobileNet	70.00	73.68	66.91	40.46	65.12	69.14
13.			10,000	UNet	83.90	86.14	81.90	67.93	81.00	83.49
14.				CNN	75.65	88.78	63.89	53.91	68.77	77.51
15.				MobileNet	68.80	74.60	63.60	38.30	64.74	69.32
16.			20,000	UNet	77.88	90.56	65.55	57.83	71.86	80.13
17.				CNN	76.55	82.14	71.12	53.54	73.42	77.54
18.				MobileNet	70.20	67.76	72.24	39.98	67.17	67.47
19.	200	Monomer	5000	UNet	87.80	79.82	94.49	75.74	92.39	85.65
20.				CNN	72.60	46.93	94.12	47.47	86.99	60.97
21.				MobileNet	68.70	55.70	79.60	36.52	69.59	61.88
22.			10,000	UNet	87.25	85.08	89.19	74.41	87.58	86.31
23.				CNN	77.85	92.59	64.64	58.99	70.11	79.80
24.				MobileNet	65.20	87.28	46.69	36.52	57.85	69.58
25.			20,000	UNet	85.62	84.78	86.45	71.24	85.87	85.32
26.				CNN	80.90	91.63	70.48	63.41	75.09	82.54
27.				MobileNet	69.30	62.50	75.00	37.83	67.70	64.99
28.	200	Dimer	5000	UNet	90.20	86.84	93.01	80.25	91.24	88.99
29.				CNN	81.70	78.29	84.56	63.04	80.95	79.60
30.				MobileNet	70.50	54.61	83.82	40.49	73.89	62.80
31.			10,000	UNet	86.35	84.97	87.58	72.60	85.97	85.47
32.				CNN	76.25	93.65	60.66	56.85	68.08	78.84
33.				MobileNet	69.10	78.84	60.38	39.69	64.06	70.68
34.			20,000	UNet	86.10	87.32	84.92	72.23	84.90	86.09
35.				CNN	82.67	91.07	74.52	66.40	77.64	83.82
36.				MobileNet	71.00	72.59	69.67	42.09	66.73	69.54
37.	300	Monomer	5000	UNet	64.20	46.05	79.41	27.14	65.22	53.98
38.				CNN	80.70	79.82	81.43	61.17	78.28	79.04
39.				MobileNet	69.80	58.33	79.41	38.77	70.37	63.79
40.			10,000	UNet	65.20	71.53	59.53	31.18	61.29	66.02
41.				CNN	80.60	92.17	70.24	63.41	73.50	81.78
42.				MobileNet	66.60	41.67	87.50	33.20	73.64	53.22
43.			20,000	UNet	65.20	71.53	59.53	31.18	61.29	66.02
44.				CNN	83.27	93.25	73.58	68.03	77.42	84.60
45.				MobileNet	69.60	67.98	70.96	38.87	66.24	67.10
46.	**300**	**Dimer**	**5000**	**UNet**	**91.60**	**96.71**	**87.32**	**83.72**	**86.47**	**91.30**
47.				CNN	85.40	83.40	87.13	70.62	84.87	84.13
48.				MobileNet	71.10	77.56	68.29	42.30	51.54	61.92
49.			10,000	UNet	88.00	83.60	92.85	76.48	92.80	87.96
50.				CNN	84.80	78.44	93.01	70.98	93.54	85.33
51.				MobileNet	69.70	65.77	74.27	39.98	74.81	70.00
52.			20,000	UNet	87.08	89.60	84.62	74.27	84.99	87.23
53.				CNN	83.63	78.24	91.14	68.43	92.50	84.77
54.				MobileNet	71.00	72.59	69.54	42.09	66.73	69.54

## Data Availability

The dataset/s that support the findings of this study are publicly available at the University of California, Santa Cruz (UCSC), Genomics Institute [https://genome.ucsc.edu/cgi-bin/hgFileUi?db=hg19&g=wgEncodeHaibMethylRrbs accessed on 22 May 2025], and the National Library of Medicine—National Center for Biotechnology Information (NCBI) [https://www.ncbi.nlm.nih.gov/nuccore/NC_000004.11/ accessed on 22 May 2025].

## Appendix A. The Pseudocode of the UNet Model Is as Follows

***Variables:***	
C:	Channels
L:	Sequence Length
M:	Methylated
U:	Unmethylated
Input: X as $Tensor\;\left(L,C\right)$	
Output: Classification labels $F_{o}=M,U$	
***Encoder Block***	
*Step 1:*	
X1 = Conv1D(X, filters=16, kernal size=3, activation=‘relu’)	
X2 = Conv1D(X1)	
X3 = MaxP1D$\left(\mathrm{pool}\;\mathrm{size}=4\right)$(X2)	
$T_{\mathrm{skipconn}}=X2$	
*Step 2:*	
X4 = Conv1D(X3,filters=32)	
X5 = Conv1D(X4)	
X6 = MaxP1D(X5)	
$T_{\mathrm{skipconn}}=X5$	
*Step 3:*	
X7 = Conv1D(X6, filters=64)	
X8 = Conv1D(X7)	
***Decoder Block***	
*Step 4:*	
Y1 = Conv1D Transpose (X8, filters=32, strides=4)	
Y2 = Concat(Y1)	
$T_{\mathrm{skipconn}}=Y1$	
Y3 = Conv1D(Y2)	
*Step 5:*	
Y4 = Conv1D Transpose (Y3 filters=16)	
Y5 = Concat(Y4)	
$T_{\mathrm{skipconn}}=Y4$	
Y6 = Conv1D(Y5)	
***Output Layer***	
*Step 6:*	
Z1 = GlobalAvgPooling1D(Y6)	
*Step 7:*	
Apply a final dense layer with activation function	
$F_{o}=\mathrm{Dense}\;\left(2,\;\mathrm{activation}=\;\text{‘}\mathrm{softmax}\text{’}\right)$(Z1)	
